# Recommendations from Transgender Healthcare Consumers in Rural Areas

**DOI:** 10.1089/trgh.2017.0052

**Published:** 2018-06-01

**Authors:** Douglas Knutson, Meredith A. Martyr, Travis A. Mitchell, Tori Arthur, Julie M. Koch

**Affiliations:** ^1^ Department of Psychology, Southern Illinois University at Carbondale, Carbondale, Illinois.; ^2^ Department of Educational Psychology, University of Minnesota, Twin Cities, Minneapolis, Minnesota.; ^3^ School of Community Health Sciences, Counseling and Counseling Psychology, Oklahoma State University, Stillwater, Oklahoma.

**Keywords:** healthcare, qualitative, rural, transgender

## Abstract

**Purpose:** Scholars indicate that rates of mental and physical health issues (e.g., substance use, anxiety, depression) may be much higher among transgender individuals relative to the general population. This disparity may be even greater for transgender individuals in rural areas. Clinical researchers suggest using affirmative therapeutic approaches and interventions to address the health concerns of transgender individuals, specifically to connect individuals with the transgender community. However, little is known about the content of information that is shared in transgender communities in rural areas.

**Method:** For this qualitative study, researchers asked transgender individuals in rural areas (*n*=10) what recommendations they would offer to other transgender individuals in rural areas regarding healthcare access.

**Results:** Results were organized into four domains: Access care, Quality control, Difficulties, and Mentorship. Within these domains, we identified 11 sub-domains: Get physical healthcare, Get mental healthcare, Provider search, Provider vetting, Treatment verification, It will be difficult, Know who you are, Believe in yourself, Move, Connect to community, and Other.

**Conclusions:** We discuss implications of our findings for healthcare provision in rural areas, and we provide recommendations for future research.

## Introduction

As with any population, transgender people rely on medical and mental health services to address physical concerns (e.g., hormone therapy, general healthcare) and psychosocial difficulties (e.g., discrimination, depression, anxiety, dysphoria).^1–3^ Throughout, we use transgender to refer to “a broad array of people” who share a “discordance between their designated sex at birth and their gender identity.”^4^ However, this reliance may be higher given that transgender populations report elevated rates of mental health concerns such as suicidality and depression, relative to their cisgender^1^ and lesbian, gay, and bisexual^1^ counterparts. Furthermore, geographic location impacts psychological distress^5^ such that transgender people in rural areas tend to report even higher levels of depression and anxiety relative to residents of urban areas.^6^

Meanwhile, transgender people may avoid seeking mental and physical health services for fear that they will be discriminated against.^7^ Though current research tends to focus on urban samples, some scholars have documented challenges faced by transgender people in rural areas when they access healthcare systems.^8^ Existing access issues are further complicated by the fact that transgender affirmative healthcare is relatively new and providers have only recently started to form visible service neworks.^9^ Though these groups of competent providers are continuing to expand, a majority of health professionals continue to report inadequate training and/or a lack of awareness of transgender healthcare guidelines.^10–12^

When studying medical training and healthcare access, researchers tend to overlook urban and rural location, as demonstrated papers that break the United States into a number of regions, but do not distinguish between urban and rural locale.^5,7,11^ Health studies that assess transgender samples also tend to include lesbian, gay, and bisexual cohorts, even though doing so obscures the differences between sexual orientation and gender identity.^13,14^ This practice further masks the uniqueness of transgender communities, but lesbian, gay, bisexual, and transgender (LGBT) research is still relied on out of necessity. In light of these limitations, what we know of transgender healthcare access in rural areas is generally stitched together from a broad range of existing information.

Given the available information about the challenges that rural transgender individuals face when they attempt to access healthcare^8^ and the historic lack of visible, competent, and well-advertised providers,^9–12^ it is no wonder that transgender people have relied on social networks and on the internet as sources of vital information.^15–17^ In fact, social networking within communities of transgender individuals is linked to resilience and to positive healthcare experiences.^16,17^ Aside from the general benefits of social support,^18^ interpersonal connections allow for the transmission of vital information and resources.^19^

Considering the benefits of social support, scholars and practitioners suggest that social components (e.g., group therapy, online connections) be included in transgender affirmative treatment.^15,20–22^ Affirmative treatment addresses the transgender person holistically and with an awareness of the lived experiences of transgender people.^7,22^ Thus, guidelines for transgender treatment providers in rural areas include recommendations to include social support components.^23^

Taking into account that scholars direct providers to encourage and cultivate interpersonal connections between transgender patients and clients, it is somewhat surprising that very little research has addressed the specific content or subject matter of the support that may be exchanged in these networks. In general, social support shares a positive relationship with health, given that people may use social connections to transmit tangible (e.g., resources, gifts) or intangible (e.g., verbal, emotional exchanges) assistance.^18,24,25^ This may be true for transgender people in rural areas where, as noted earlier, transgenders face disproportionately high rates of health concerns.

As noted by Wanta and Unger, many gaps exist in the transgender health literature that may be addressed by using qualitative methodologies.^26^ One such gap is our understanding of the actual health-related information and assistance that may be provided through peer-to-peer networks of transgender people in rural areas where health concerns and difficulties with access may be most salient. In an effort to fill this gap in the literature and to provide greater insight for individuals who wish to provide competent, affirmative therapy, we asked a sample of transgender people in rural areas to answer the question, “What recommendations would you make to future generations of transgender or gender nonconforming individuals in rural areas who may seek health care?” We were guided by the research question, “What guidance may be offered and circulated within transgender communities in rural and remote areas of the United States?”

## Method

We used Consensual Qualitative Research (CQR) analysis^27^ to explore and organize participant responses to a question about recommendations they would offer to other transgender people in rural areas in search of healthcare. Our focus on a homogenous sample,^28^ in terms of both rurality and transgender identity, yielded rich information for in-depth study and inductive analysis.^28,29^

### Participants

We recruited a purposive convenience sample of 10 individuals, 18 years old or older, who identified as transgender or gender diverse and who reported living in rural or remote areas of the United States. A majority of our participants identified as White (*n*=7) and the rest identified as either multi-racial (*n*=2) or Alaskan native (*n*=1). Regarding gender identity, the majority of our participants identified as female (*n*=5), the second largest group identified as trans men (*n*=2), and the remaining participants identified as male (*n*=1), trans female (*n*=1), and non-binary (*n*=1). Our participants were diverse in terms of sexual orientation (Table 1), and participant ages ranged from 23 to 59 years old (*M*=36.2).

**Table 1.** **Demographic Information**

*Item*	n
Ethnicity	
White	7
Multi-racial	2
Alaskan native	1
Gender identity	
Female	5
Male	1
Trans man	2
Trans female	1
Non-binary	1
Sexual orientation	
Pansexual	4
Bisexual	2
Heterosexual	1
Polysexual	1
Intersex	1
Not sure	1

***Item***	***Range (M)***
Age (in years)	23–59 (36.2)

All participants endorsed that they were from a rural area or small town when they completed the demographic form. Given the qualitative design of the study and the inherent subjectivity in what may be considered *rural*,^30^ we included participants who self-identified as rural residents both in their responses to the demographic form that was used to screen potential participants and in the semi-structured interview. During the interview, participants described their rural locations in terms of the limited number (or absence) of stoplights or stop signs in the town nearest them, the duration of their commute to the nearest urban area (in hours), or the proximity of their nearest neighbor (in miles). Considering that one's identity as a rural resident speaks to the interpersonal, cultural values under examination in this study, self-report was an adequate measure of rurality.^30^

### Procedures

We recruited participants through Facebook groups for transgender individuals, LGBT organizations, and professional list-serves that reach both state and national transgender populations. Prospective participants contacted the primary investigator via email to express interest in participating. Interested individuals were then emailed a link to an informed consent document, demographic questionnaire, and interview scheduling matrix. The consent document informed participants that participation was voluntary and, at the bottom of the form, participants were asked to endorse their agreement to participate by checking a box. Semi-structured interviews lasting one hour to one hour and thirty minutes were conducted via phone or video conferencing.

### Measures

Our 15-item semi-structured interview (Table 2) was assembled based on our review of existing literature regarding healthcare access among transgender people^23^ as well as LGBT access literature to supplement the sparse information that was available regarding only transgender populations.^26^ The sequence and structure of questions built from general to more specific.^27,29^ The interview included questions about transition, location, healthcare experiences, personal reflections, and recommendations for providers and individuals. After asking each question in the protocol, we followed up with requests for specific examples, anecdotes, and stories.

**Table 2.** **Semi-Structured Interview Script**

1. Before we start, I (we) want to make sure that we are addressing you correctly. Can you please tell me (us) your chosen name? What pronouns would you like us to use?
2. Great. Can you tell us briefly about your gender identity?
3. Are you transitioning or do you plan to do so in the future? If so, what is your ideal timeline for transition?
4. As you know, we want to learn more about trans people living in rural areas. We would like to get a better sense of where you live. Please tell us just a little bit about the rural area in which you live.
5. As a trans person in a rural area, do you think your experiences with healthcare have been different than the experiences of cis people in rural areas? Could you please discuss some of these differences?
6. Do you think your experience with healthcare would be different if you lived in a city? If so, please explain how.
7. In your view, what are your most pressing healthcare needs?
8. Do you think that being in a rural area contributes to any health-related hardships you may experience? If so, could you please discuss these hardships and how your rural location increases them?
9. Are there benefits to your health of being transgender in a rural community/location? If so, could you please discuss these benefits?
10. Are there any other transgender people in your community and, if so, what is the quality of your connection with these people? (Possible prompts: how far away/accessible)
11. Have you accessed health services in an urban area in the past? If so, what are your experiences with healthcare providers in rural locations as compared to your experiences with urban healthcare providers?
12. Did you seek out specific providers to meet your healthcare needs as a trans person? If so, how did you locate resources, information, and healthcare providers given that you are in a rural area?
13. What are recommendations you would make to healthcare providers who wish to provide services for rural transgender and gender nonconforming populations (e.g., quality and availability of services)?
14. What advice or recommendations would you make to future generations of transgender or gender nonconforming individuals in rural areas who may seek healthcare?
15. Do you have any additional thoughts or information we should know about in regard to your experience in a rural area?

After we completed each interview, we had the resulting audio file transcribed verbatim by an Health Insurance Portability and Accountability Act (HIPAA) compliant transcriber. We then checked each transcript for accuracy by a research team member and, during this process, team members ensured that the transcripts were thoroughly deidentified.^27,29^ We tracked the deidentified transcripts by using randomly assigned identification numbers. This study was reviewed and approved by the Institutional Review Board at Oklahoma State University. Our analyses for this study focused only on responses to the question, “What advice or recommendations would you make to future generations of transgender or gender nonconforming individuals in rural areas who may seek healthcare?”

### Analysis

Our team used several files to organize the data, including a domain table, transcript files for each interview, a document with interview data organized by domains, and a quote document.^29^ The research team synthesized domains inductively based on the first transcript and, where disagreement existed, we discussed our perspectives until we reached consensus.^27^ Then, we applied domains formulated during analysis of the first transcript and to all subsequent transcripts, through group consensus. Throughout the coding process, the initial domain list was revised, expanded, and condensed to address the information provided by each new interview.^29^ After applying domains to the sixth transcript, the research team submitted all data and the domain list to the auditor.^27^ The research team worked with the auditor to respond to the auditor's comments and to revise domains as appropriate. Finally, we applied domains to the remaining four transcripts.

One major content area resulted from the 14th semi-structured interview question, “What advice or recommendations would you make to future generations of transgender or gender nonconforming individuals in rural areas who may seek healthcare?” The data obtained from this question were dissimilar from the rest of the results and, therefore, we separated these data from the other content and placed them under a temporary heading, *recommendations for individuals.*

The primary investigator pasted all of the *recommendations for individuals* content into one document and organized the data thematically, creating domains for each thematic group. The research team and auditor provided further analysis and feedback about the reorganized data. The resulting domains and sub-domains are listed in Table 3 along with the frequencies at which these domains and sub-domains appeared in each interview response set.^29^

**Table 3.** **Domains and Sub-Domains with Frequencies by Response Set**

	*Interview response set*									
*Domain sub-domain*	*A*	*B*	*C*	*D*	*E*	*F*	*G*	*H*	*I*	*J*
Access care										
Get physical healthcare				X	X					
Get mental healthcare						X		X		
Quality control										
Provider search							X	X		X
Provider vetting		X				X			X	
Treatment verification	X					X				
Difficulties										
It will be difficult	X	X	X		X					X
Know who you are	X					X	X	X		
Believe in yourself	X				X					X
Mentorship										
Move			X		X			X		
Connect to community				X	X				X	X
Other	X		X					X		

### Researchers and reflexivity

For the formation of our research team, we followed CQR recommendations regarding size, consensus building, inclusion of an auditor, and identification of biases before and throughout the data analysis process.^27^ Our final research team was composed of five members: a gay, male doctoral student; a pansexual, female doctoral student; a heterosexual, male doctoral student; a queer, female doctoral student (the auditor); and a queer, female faculty member. All research team members identified as White and cisgender, and they were affiliated with American Psychological Association (APA) Accredited Counseling Psychology programs in the Midwest and Mid-South. Though all researchers identified as cisgender, the auditor had extensive experience providing therapy services to transgender clients.

Before the first research team meeting, research team members read information on phenomenological, exploratory qualitative methodologies, including excerpts from the CQR manual.^27^ Members met to discuss preexisting assumptions about transgender and/or gender diverse individuals as well as expectations regarding possible outcomes of the study.^27^ Members shared the assumption that fewer resources are available in rural areas and that rural participants may face more challenges than urban dwellers. Overall, the research team shared a common spirit of advocacy. The research team kept themselves and other group members aware of the impact of bias throughout the data analysis process.^27^

## Results

Four domains took shape as we reviewed our participants' recommendations for other transgender people in rural areas. These domains were: Access Care, Quality Control, Difficulties, and Mentorship. A few participant responses did not fit under a specific domain and were, therefore, collected under an independent sub-domain labeled *other*.

We provide quotes throughout our results in an effort to give a more direct voice to our participants. We removed verbal fillers (e.g., um, uh, like) to increase the accessibility of the quotes and we used “they/them/their” pronouns to deidentify the content as well as to remain gender neutral when we refer to our participants.

### Access care

Content in this domain provides insight into the ways that rural individuals may encourage each other to obtain both mental and physical health services. Our participants expressed awareness that some hormones and medications are available online and/or outside of the healthcare system. However, our participants encouraged the use of caution when accessing medications and other interventions without the oversight of a licensed professional.

This domain contained two sub-domains. In the first, *get physical healthcare*, participants encouraged others to access health services even if doing so felt frightening. One participant, for example, expressed concern for transgender men and women who may not undergo regular physicals that screen for cancers that may affect individuals who share the gender that these transgender men and women were assigned at birth. The participant reported worrying that worry about discrimination was endangering the lives of transgender people in rural areas. One of our other participants stated, “I think my biggest advice to future transgender and non-binary people is to not be afraid of health care…and that it's really important to take care of your health.”

Participants also encouraged others to *get mental healthcare*. One of the participants stated that the same mental health issues (e.g., depression, anxiety) a person faces before transition may be the same issues the person faces after transition. This participant further clarified their thoughts by stating, “transition doesn't fix everything.” Another participant communicated a value for mental healthcare by saying, “Not everybody can afford to go to therapy, but go when you can…I mean that's something that does really need to be watched.” The individual went on to highlight that attention from a mental health provider may reduce the risk for suicide among transgender people in rural areas.

### Quality control

The *quality control* domain included insight into ways that participants obtained health-related information. Our participants provided insight into the process by which transgender communities may search for providers, vet providers, and/or verify that the treatments they are receiving are appropriate. The general message under this domain was well framed by one participant who said, “Do your homework. Do your research.” This mantra seemed to underlie many of the suggestions made by our participants. Overall, participants suggested that the burden of quality control for transgender healthcare services may fall on the person who is accessing those services. As one participant admonished others, “You have to do, you have to keep fighting and do what it takes to get to these doctors.”

The first sub-domain within *quality control* was *provider search*. Aside from standard ways of searching for doctors and therapists such as reviewing in an insurance provider's website, our participants endorsed more social ways of locating healthcare professionals. For example, one participant said, “…do rely on google, rely on Facebook, find out from people you trust and if you don't know anyone, find someone [you trust in the community].” This participant and others emphasized the need to rely on other community members for reliable referrals.

Under the *provider vetting* sub-domain, participants encouraged others to not only trust referrals from community members but also verify that treatment providers will be able to meet the needs of each individual. Our participants highlighted how disappointing it can be to seek services from a provider, to spend time and money on services, and then to find out that the provider is not willing or able to render the treatment sought by the transgender patient or client. Participants also noted that the attitudes and reactions of front office staff, as the first point of contact with a provider's office, are the data on which prospective transgender patients or clients in rural areas base their decisions about whether or not to see a provider. They offered reminders to themselves and others that outpatient treatment includes the freedom to fire doctors and other providers. One individual stated, “You have a right to question the doctor…to basically interview them and see if that's actually who you want to see.”

Lastly, under the *treatment verification* sub-domain, participants encouraged others to learn about best practices both before and during treatment.. They spoke about the importance of remaining critical of both physical and mental health treatments, rather than assuming that recommendations or diagnoses were correct because they were made by a licensed health provider.

### Difficulties

This domain represented participant reflections on the additional burden of accessing healthcare in rural areas. *Difficulties* is characterized by a quote from one individual who stated, “I guess transgender people [are] trying to make themselves more visible. And that's kind of like putting a target on your back.” As participants discussed the dangers and issues related to healthcare access, they explored ways to optimize success in such an adversarial environment. Three sub-domains reflected participants' advice to other transgender individuals regarding healthcare encounters; *it will be difficult; know who you are; and believe in yourself.*

In general, participants acknowledged the difficulty involved in accessing healthcare in rural areas for transgender-identified individuals. One individual stated plainly, “I do think it is going to be hard [for others], but keep up the fight.” We address the barriers and issues associated with healthcare access in rural areas more comprehensively elsewhere because the full data set included reflections on negative experiences with providers,^8^ but it is important to acknowledge that this difficulty with access is communicated within rural transgender communities as well.

Within the *know who you are* sub-domain, participants discussed the importance of not only learning about healthcare systems but also simultaneously exploring one's own identity in the process. As a way to respond to the difficulties faced in rural areas, participants discussed the importance of coming out to oneself and self-discovery as elements in the process of seeking healthcare, particularly highlighting benefits of coming out earlier in life. One participant said, “Before you know what health care you need and what health care to seek, you kind of have to know who you are.” Another individual suggested that diagnoses and care may help with self-understanding when they stated, “I really think a diagnosis might have helped me a long way in my acceptance of myself.”

The *believe in yourself* sub-domain added an additional dimension to self-discovery. Not only did participants admonish each other to explore their identities, but they also encouraged self-trust. These participants stressed the importance of bravery, confidence, and self-reliance. One individual stated simply, “I mean, you know, believe in yourself…” Another participant said, “Develop a thick skin and develop some bravery. That's what I can think of.” As they discussed the importance of self-trust, these participants emphasized their pride in their community and in their identities.

### Mentorship

Our final domain, *mentorship* included content that could best be described as wisdom dispensed from experienced advisors. Unlike the content described earlier, the statements in the *mentorship* domain were more interpersonal in nature and were drawn from aspects of the individual's social experiences as a transgender person in a rural area. We further divided this domain into two sub-domains, *community* and *move*.

Regarding community engagement, several of our participants discussed the importance of finding other transgender individuals or groups of transgender people either locally or on the internet. In general, there was a recognition among participants that the internet may be an indispensable source of connection for those who have access to it. Participants highlighted the importance of knowing even one person who can give sound advice or offer support and they noted other benefits of mentors and contacts such as the ability to pool resources. For example, one participant stated, “…talk to people, do as much as you can to make connections in your own community and find out who is friendly…” Another individual offered, “Find community support if you can. If you can't, create it.”

Though few (if any) of our participants were able to move to an urban area themselves, some recommended moving out of rural areas. Again, these data are addressed in more detail elsewhere,^8^ but in the context of this article, it is important to note that some of our participant members stated that they believed urban areas were better equipped than rural areas to meet their needs. These sentiments were not unanimous, but the majority of participants expressed a belief that cities are better equipped to meet the healthcare needs of transgender people.

## Discussion

The goal of this project was to explore the content of health-related information and assistance that may be provided through peer-to-peer networks of transgender people in rural areas. Given that scholars and researchers encourage providers of affirmative services to cultivate and encourage connections with transgender communities and groups,^20–22^ we sought to offer insight into some of the information and advice that may be provided in informal rural networks. Four main domains and 11 sub-domains surfaced during our inductive, exploratory analysis of participant responses regarding access to healthcare. Results may assist healthcare professionals (e.g., physicians, counselors, and psychologists) who seek to provide quality and informed services for transgender individuals.

Before delving into the implications of our data for healthcare providers and for future research, we should note that the participants who offered these perspectives were speaking to other transgender people in rural areas within the context of a broader study on healthcare. First and foremost, our findings include empowering statements about communal resilience, pride, and solidarity. To the degree that we apply our findings to healthcare provision, we may run the risk of eavesdropping. Recognizing this, we hope that our discussion does justice to our participants and meets the overall goal of improving services.

### Implications for care

Researchers have conducted studies of online resource use,^15^ of the process by which transgender patients choose a surgeon,^9^ and of ways that fear of mistreatment reduces the likelihood that transgender people will access healthcare in the first place.^7^ Less is known about how this knowledge is transmitted within peer-to-peer social support networks rural communities where transgender individuals tend to experience disproportionate exposure to discrimination and more psychological distress.^5,6^ For example, issues with medical training have been well documented by researchers,^11,12^ but investigators know less about how these dynamics impact perceptions of healthcare within rural transgender social networks.

Despite their knowledge that they may encounter prejudice, mistreatment, and other issues while accessing care, our participants, nevertheless, expressed commitment to pursuing care. This tenacity is remarkable in the context of so much evidence that healthcare settings are oppressive spaces for transgender people.^31,32^ Though fear may impact transgender people on an individual level,^7^ our participants continued to see the value for health services and they were inclined to recommend treatment to their peers. When clients or patients become connected with other transgender community members, they may benefit from this encouragement. To the degree that this is the case, individuals who are connected with peers may be more likely to adhere to treatment.

Researchers have highlighted that connections to transgender communities may foster resilience.^16^ Our study further illustrated ways this positive impact may be fostered by offering insight into the ways that transgender people in rural areas may encourage each other to embrace their identities, to see their own value, and to use their self-awareness as a source of motivation to seek care despite the inherent challenges. Connecting individuals to therapy groups and online networks not only equips them with sources of information but also carries the added benefit of fostering pride, resilience, and identity formation.

Furthermore, our study provides insight into the degree to which transgender people in rural areas may share knowledge about the limitations and ineffectiveness of specific providers. Providers themselves may be unaware of the degree to which transgender clients and patients are aware of the lack of competence among providers and the astuteness with which rural transgender people are able to assess a provider's competence. Our participants noted that their connection to other transgender people in their rural area provided them with a wealth of information about the local providers. One implication of this dynamic is that a single negative or transphobic encounter with an office staff-person could place a provider's office on a communally maintained list of prohibited providers. Thus, providers who wish to provide transgender affirmative services may benefit from cultivating relationships, not only with individuals but also with the community itself.

Our participants also encouraged others to be aware that, as patients and clients in outpatient settings, they are free to question and fire their healthcare providers. They regularly indicated that they seek out sources of information about best practices on the internet and that they share this information with each other and with their physicians and therapists. Given that transgender people in rural areas may have access to vast networks of other community members, they may often be more informed and up-to-date on current interventions than their providers. Given this reality, providers who operate with humility and openness to feedback or direction may work most effectively with transgender individuals in rural areas.

Participants in our study disclosed a general bias against rural providers that is supported in current research literature indicating regional differences in quality of care.^5^ Although this dynamic has been highlighted in existing literature,^8^ our findings provide context for how widespread this assumption may be in rural transgender communities. As providers in rural areas obtain more competence in transgender healthcare, this perception may change. At present, however, providers in rural areas may have to try harder to counter this perception in advertising and outreach.

### Limitations

This study was qualitative and has limitations relative to quantitative research such as comparatively small sample sizes and limited generalizability. Our results should not be generalized to the entire rural transgender population and should be approached with an awareness of the limitations inherent in data obtained from such a small sample. In addition, our research team was composed of all cisgender researchers. Our auditor was a person with extensive experience providing therapeutic services to transgender people in rural areas, and we worked hard to monitor our biases and assumptions. Nevertheless, our common identities may have impacted our results.

The research team experienced difficulty finding and recruiting transgender individuals in rural and remote areas, which resulted in relying heavily on Facebook groups for regional transgender communities in the United States. In addition, interviews were conducted over internet-based video conferencing software and/or over the phone and our informed consent, demographic survey, and scheduling form were all hosted online. Thus, prospective participants who have little to no access to technology, internet, and reliable phone service may have been excluded. Thus, our findings may represent the perspectives of people who connect with community online and not of individuals without internet access. Furthermore, to the degree that we accessed participants through groups that serve lesbian, gay, and bisexual individuals, we may have inadvertently recruited more participants who were diverse in terms of sexual orientation.

Lastly, the research team was composed of mental health professionals and scholars, which may have created a barrier to participation for some individuals who may have previously experienced trauma involving healthcare providers.

## Conclusions

This study is one of the few that provides insight into the actual characteristics of information exchanged in social networks of transgender people in rural areas. Understanding the content of interpersonal exchanges in transgender communities may support the creation of more effective health services and community-building initiatives. Additional research is needed to assess dimensions of community building and shared knowledge in rural transgender communities that reach beyond healthcare utilization and access.

## Abbreviation Used

CQR: consensual qualitative research
